# Tissue Infiltrating Immune Cells as Prognostic Biomarkers in Endometrial Cancer: A Meta-Analysis

**DOI:** 10.1155/2020/1805764

**Published:** 2020-01-28

**Authors:** Fang Guo, Yishan Dong, Qingqing Tan, Jing Kong, Bin Yu

**Affiliations:** Department of Medical Genetics, Changzhou Maternity and Child Health Care Hospital Affiliated to Nanjing Medical University, Changzhou 213003, China

## Abstract

**Background:**

The association between tumour-infiltrating immune cells and the prognosis of endometrial cancer (EC) is controversial due to the smaller sample sizes and limited statistical power of the extant studies. We carried out a meta-analysis of the relationship between tumour-infiltrating immune cells and EC survival outcomes.

**Methods:**

A literature search in multiple databases was carried out up to December 2019. Pooled hazard ratio (HRs) and 95% confidence intervals (CIs) were calculated by the *Z*-test to assess the association between infiltrating immune cells and overall survival (OS), progression-free survival (PFS), relapse-free survival (RFS), disease-specific survival (DSS), and disease-free survival (DFS). A subgroup analysis was performed based on the localisation of immune cells in tumour parenchyma or stroma, immune markers, and the International Federation of Gynecology and Obstetrics stage. Heterogeneity and publication bias between studies were evaluated by Cochran's *Q*-test and Egger regression test, respectively.

**Results:**

Seventeen studies were included in the analysis. The pooled HR of OS, PFS, DSS, and DFS indicated that a high CD8^+^ T cell density was associated with a favorable prognosis in EC patients. A significant relationship was found between a high density of CD45RO^+^ T cells and a favorable OS in EC patients, but the FoxP3^+^ T cell density was not associated with either OS or RFS. A high TAM density was associated with a worse PFS. However, a sensitivity analysis indicated that the findings of PFS and DSS in CD8^+^ T cell and PFS in TAM were not robust results.

**Conclusion:**

This is the first meta-analysis of the relationship between tumour-infiltrating immune cells and the prognosis of EC. High CD8^+^ and CD45RO^+^ T cell densities in tumours were associated with favorable outcomes in EC patients.

## 1. Introduction

Endometrial cancer (EC) is a gynaecologic malignancy and the sixth leading cause of death in females; the prevalence of EC is increasing worldwide [1]. Despite treatment, such as surgical resection, chemotherapy, and/or radiotherapy, the survival rate of EC is low due to its propensity to proliferate and metastasize. Immunotherapy is effective for EC, and immune cells, which infiltrate in the tumour microenvironment, play important roles in tumour progression and metastasis [2].

The tumour microenvironment comprises diverse types of immune cells, including dendritic cells (DCs), natural killer (NK) cells, mast cells, macrophages of the innate immune system, and lymphocytes of the adaptive immune system [3]. Each immune cell has a different effect on tumour progression. Some immune cells participate in antitumour immunity and tumour surveillance, whereas others moderate the inflammatory response and promote angiogenesis and tissue remodelling, accelerating tumour growth. CD8^+^ T cells recognise tumour antigens and differentiate into cytotoxic T lymphocytes, which attack and kill tumour cells. EC with a high intraepithelial CD8^+^ T cell density has also been reported to have more favorable outcomes. Similarly, a high density of DCs, which perform antigen presentation and may activate tumour antigen-specific T lymphocytes, and CD45RO^+^ T cells, a type of memory T-lymphocyte that recognises tumour antigens, are also associated with enhanced survival of patients with EC. Tumour-associated macrophages (TAMs) release a series of proangiogenic factors and cytokines, promoting tumour invasion, growth, metastasis, and angiogenesis. A high density of TAMs or FoxP3^+^ regulatory T cells is thought to be associated with an unfavorable EC prognosis.

Prior studies have yielded inconsistent results for the impact of immune cells on EC outcomes due to small sample sizes or the use of different prognostic indices. For example, some studies have found that patients with a high CD68^+^ TAM density had worse relapse-free survival (RFS) or overall survival (OS) than those with a low TAM density [4, 5], and others have shown that a high CD163^+^ TAM density was associated only with RFS [6] or was not associated with the survival outcomes of EC patients [7, 8].

We conducted a meta-analysis of the effects of infiltrating immune cells on the prognosis of EC. Moreover, we carried out subgroup analyses according to immune cell localisation, immune markers, and the International Federation of Gynecology and Obstetrics (FIGO) [9] stage to identify sources of variation.

## 2. Materials and Methods

### 2.1. Search Strategy and Inclusion Criteria

The meta-analysis was designed and implemented according to the recommendations of the Preferred Reporting Items for Systematic Reviews and Meta-Analyses (PRISMA) statement [10, 11] (PRISMA 2009 checklist is shown in Supplementary Materials ([Supplementary-material supplementary-material-1])). We conducted a literature search in the PubMed, EMBASE, Scopus, and Cochrane databases using the following keywords: “endometrial neoplasms or endometrial cancer or endometrial carcinoma or endometrial tumour” and “immune cell” or with either “tumour-associated macrophages or TAMs or tumour-infiltrating macrophages or intratumoural macrophages,” “mast cell,” “dendritic cell,” “NK cell or natural killer cell,” “lymphocyte or tumour-infiltrating lymphocytes or regulatory T cell or CD3^+^ T cell or CD4^+^ T cell or CD8^+^ T cell or FoxP3^+^ T cell or CD57 T cell or CD45RO^+^ T cell,” and “B cell.” The final search was performed on December 13, 2019. The references of the retrieved studies were also searched. Only articles written in English were included. The inclusion criteria were as follows: (1) studies of the correlation between tumour-infiltrating immune cells and EC survival outcomes, (2) the detecting method which was immunohistochemistry, and (3) hazard ratios (HRs) and 95% confidence intervals (CIs) which were provided or could be reconstructed.

### 2.2. Data Extraction and Quality Assessment

Two researchers reviewed eligible studies independently, and all researchers resolved disagreements by discussion. Extracted data included the first author's surname, year of publication, ethnicity of the subjects, number of samples, age of the subjects, immune cell types, follow-up duration, TNM stage, threshold of immune-cell density, localisation of immune cells in tumour tissue, and HRs of survival outcomes. The survival outcomes were OS, progression-free survival (PFS), RFS, disease-specific survival (DSS), and disease-free survival (DFS). OS was calculated from the date of surgery to that of death or the final follow-up. PFS was calculated from the date of surgery to that of progression, recurrence, death, or the final follow-up. RFS was calculated from the date of surgery to that of recurrence or the final follow-up. Patients were considered relapse free if they died of other diseases and there was no evidence of EC relapse. DSS was calculated from the date of diagnosis to that of death due to EC or metastasis, or the final follow-up. DFS was calculated from the date of diagnosis to that of recurrence date or death due to EC.

Two authors independently evaluated the quality of eligible studies using the Newcastle–Ottawa Scale (NOS) [12], and all researchers resolved disagreements by discussion. The standard for evaluation was based on patient selection, comparability, and outcomes.

### 2.3. Statistical Analysis

STATA software was used for statistical analysis (ver. 12.0, Stata Corp, College Station, TX, USA). The HRs (95% CI) were extracted directly from eligible studies. Engauge Digitizer software was used to reconstruct the HR and 95% CI from studies that provided only Kaplan–Meier survival curves [13, 14]. Because multivariate analysis was deemed more powerful than univariate analysis [15], multivariate analysis data were used if both univariate and multivariate analyses were performed. In addition, several studies calculated the HR and 95% CI using different references. We specified one uniform reference (low cell density) and recalculated the HR and 95% CI of any nonuniform studies. The pooled HR and 95% CI were calculated to evaluate the relationship of immune cells with the prognosis of EC. A pooled HR of <1 (no overlapping 95% CI) was considered to indicate a good prognosis. We used a random effects rather than a fixed effects model because the former takes into account heterogeneity between studies [16]. Heterogeneity between studies was assessed by Cochran's *Q*-test and was visualised using forest plots. Additionally, we performed subgroup analyses when there were at least two studies or two groups included according to immune markers, the localisation of immune cells in tumour tissue, and the FIGO stage. The localisation of immune cells was classified as follows: (a) tumour nest, cells infiltrating the tumour nest or closely connected to tumour cell; (b) tumour hotspot, cells located in the center of the necrotic area; (c) tumour stroma, cells infiltrating the tumour stroma; and (d) tumour margin, cells infiltrating the invasive margin [17]. Immune cells infiltrating tumour nests or hotspots were regarded as intraepithelial immune cells, and those in the tumour stroma or margin were considered stromal immune cells [18]. We performed a sensitivity analysis by removing each study in sequence and calculated the stability of the pooled HRs of the remaining studies. The Egger regression test was conducted to examine potential publication bias.

## 3. Results

### 3.1. Literature Search and Characteristics of Eligible Studies

**Figure 1:**
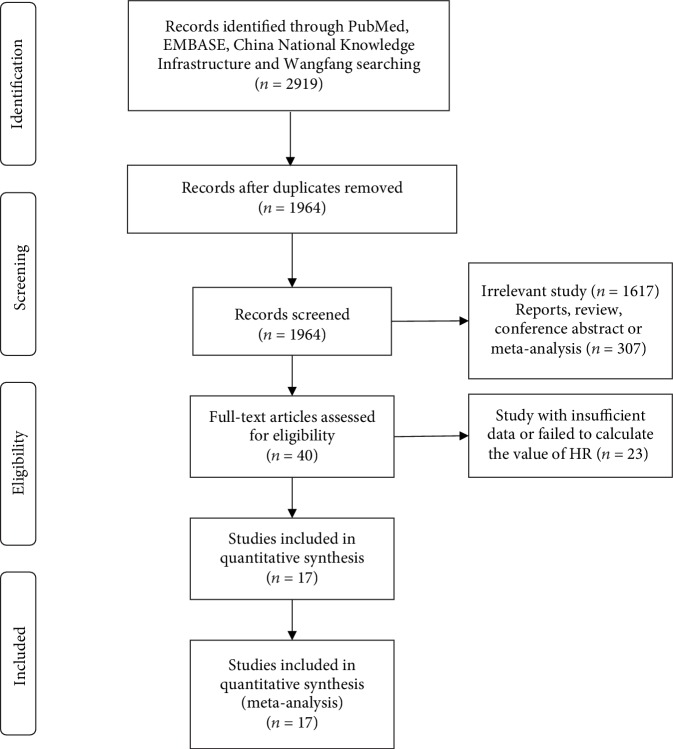
Selection of studies for inclusion in the meta-analysis.

We retrieved 2,919 articles, of which 17 [4–8, 17–28] were ultimately included in the meta-analysis. One immune cell type will be analysed when there were at least two studies that reported the same prognostic index. Studies are all shown in Table 1, but data of the studies of the same populations were only extracted once.  Figure 1 shows the process of study selection. The NOS scores of all eligible studies were >5. The characteristics, HRs, and 95% CIs of the study are shown in Table 1 and [Supplementary-material supplementary-material-1].

### 3.2. CD8^+^ T Cell

**Figure 2:**
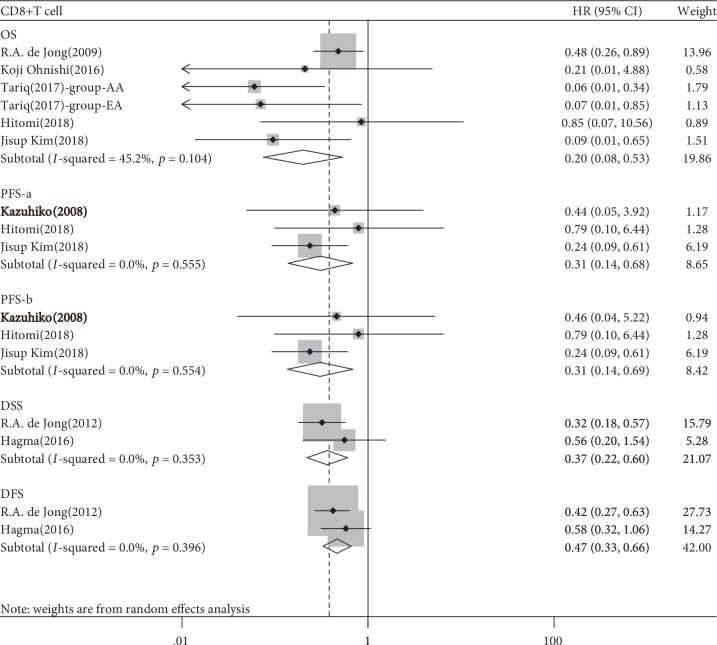
Forest plots of the relationship between tumour-infiltrating CD8^+^ T cell and survival prognosis of EC patients.

Five studies involving 740 patients were evaluated for the association between CD8^+^ T cells and OS, three studies of 630 patients for PFS, and two studies of 673 patients for DSS and DFS. The pooled HR for OS indicated that a high CD8^+^ T cell density in tumour tissues was associated with a favorable prognosis of EC (HR 0.201, 95% CI 0.076-0.533, *p* = 0.001, Table 2, Figure 2). The subgroup analysis yielded a significant association between a high CD8^+^ T cell density in intraepithelial or stromal tumour tissue and a favorable OS (intraepithelial, HR 0.320, 95% CI 0.120-0.849, *p* = 0.002; stromal, HR 0.064, 95% CI 0.016-0.253, *p* = 0.001; Table 3, [Supplementary-material supplementary-material-1]). We obtained a similar result for PFS (a study by Ino et al. [18] reported two Kaplan–Meier curves of PFS according to the immune cell localisation in tumour tissue; we calculated the HR and 95% CI of that study separately: HR-a/b 0.308/0.306, 95% CI 0.139-0.683/0.137-0.687, *p* = 0.004), DSS (HR 0.366, 95% CI 0.222-0.604, *p* < 0.001), and DFS (HR 0.466, 95% CI 0.329-0.660, *p* < 0.001; Table 2). No subgroup analysis according to the FIGO stage was conducted because all the included studies did not report the detailed stages of participants.

### 3.3. FoxP3^+^ T Cell and CD45RO^+^ T Cell

**Figure 3:**
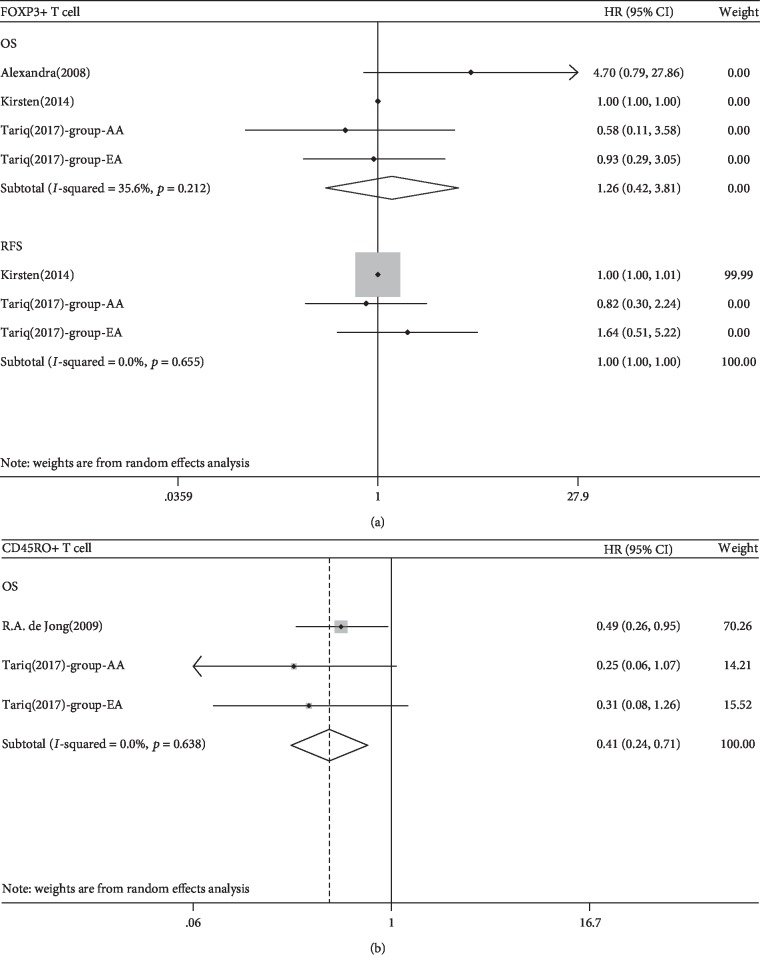
(a) Forest plots of the relationship between tumour-infiltrating FoxP3^+^ T cell and survival prognosis of EC patients. (b) Forest plots of the relationship between tumour-infiltrating CD45RO^+^ T cell and survival prognosis of EC patients.

Three studies involving 352 patients were evaluated for the relationship between FoxP3^+^ T cells and OS and two studies of 273 patients for PFS. We found no associations between FoxP3^+^ T cell density and OS or RFS (*p* > 0.05; Tables 2 and 3, Figure 3(a) and [Supplementary-material supplementary-material-1]). No subgroup analysis according to the FIGO stage was conducted because all the included studies did not report the detailed stages of participants.

For CD45RO^+^ T cells, two studies involving three groups of 478 patients were evaluated for the association between CD45RO^+^ T cell density and OS. A significant relationship was found between a high density of CD45RO^+^ T cells and a favorable prognosis (HR 0.415, 95% CI 0.241-0.714, *p* = 0.001; Table 2, Figure 3(b)). Intraepithelial or stromal tumour-infiltrating CD45RO^+^ T cells were associated with a favorable prognosis in EC patients (*p* = 0.012 and 0.031, Table 3, [Supplementary-material supplementary-material-1]). No subgroup analysis according to the FIGO stage was conducted because all the included studies did not report the detailed stages of participants.

### 3.4. TAM

**Figure 4:**
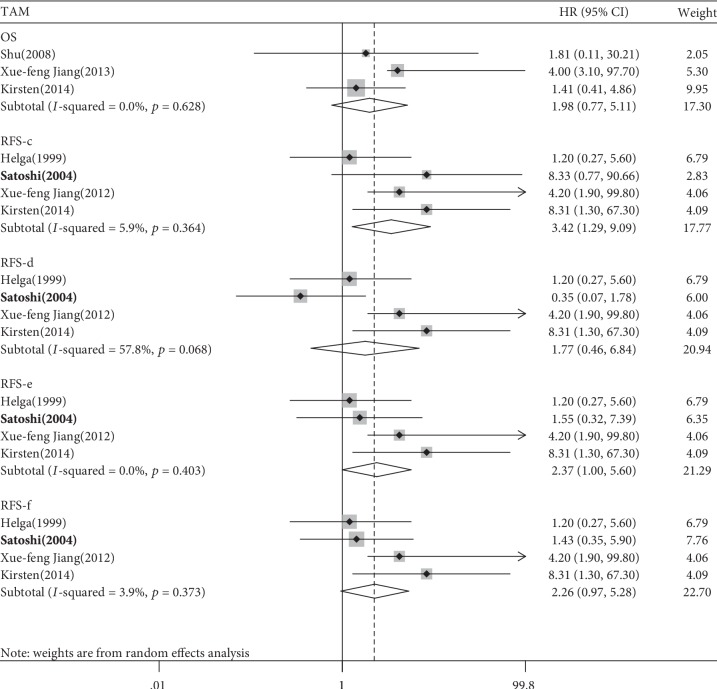
Forest plots of the relationship between tumour-infiltrating TAM and survival prognosis of EC patients.

Three studies of 425 patients for OS and four studies of 479 patients for RFS were analysed to investigate the relationship of TAMs with outcomes. No relationship was found between a low TAM density and OS neither in the pooled HR analysis nor in the subgroup analysis (pooled HR = 1.985, 95% CI 0.771-5.113, *p* = 0.156; Tables 2 and 3, Figure 4 and [Supplementary-material supplementary-material-1]).

Ohno et al. [17] provided four Kaplan–Meier curves for RFS according to histological localisation of TAM [17] (c, tumour hotspot; d, tumour nest; e, tumour margin; f, tumour stroma). The pooled HR and 95% CI were calculated based on the four HRs (c-f, [Supplementary-material supplementary-material-1]). A significant relationship between a high tumour-infiltrating TAM density and poor PFS was found for two of the four pooled HRs (HR-c 3.423, 95% CI 1.289-9.094, *p* = 0.014; HR-e 2.369, 95% CI 1.003-5.598, *p* = 0.049; Table 2 and Figure 4). The subgroup analysis according to immune markers showed that only the density of CD163^+^ TAMs was significantly associated with a worse PFS (HR 8.310, 95% CI 1.030-67.300, *p* = 0.035; Table 3, [Supplementary-material supplementary-material-1]). No subgroup analysis according to the FIGO stage was conducted because all the included studies did not report the detailed stages of participants.

### 3.5. Publication Bias and Sensitivity Analyses

There was no obvious publication bias. A sensitivity analysis showed that the pooled HRs were robust, with the exception of the association of CD8^+^ T cells with PFS and DFS and of TAMs with RFS ([Supplementary-material supplementary-material-1]).

## 4. Discussion

To our knowledge, this is the first meta-analysis of the relationship of tumour-infiltrating immune cells with the prognosis of EC. A high CD8^+^ and CD45RO^+^ T cell density in tumours was associated with favorable outcomes in EC patients. A high TAM density was associated with a worse outcome of EC, but a sensitivity analysis showed that this result was not robust.

It has been reported that advanced EC patients have been potentially eligible for immunotherapy testing in over 50 clinical trials [1]. PD-L1 is an immune checkpoint in EC that blocks the activation of T cells [29]. CD8^+^ T cells are activated by tumour antigens presented by MHC class I molecules, and exert direct cytotoxic and antitumour effects in the tumour microenvironment. Several clinical studies have shown that a high density of CD8^+^ T cells was correlated with longer survival [19, 20, 24, 25], but other studies have found no such relationship [18, 21–23]. In the present meta-analysis, the pooled HRs of the ten included studies indicated significant associations between a high CD8^+^ T cell density and the OS, PFS, DSS, and DFS of EC patients. However, the sensitivity analysis indicated that the PFS and DSS results were not robust. In a subgroup analysis according to the localisation of CD8^+^ T cells, patients with a high density of CD8^+^ T cells in the intraepithelial region of the tumour were more likely to have a good PFS than those with a high density of CD8^+^ T cells in the tumour stroma. Result in DSS failed to find the heterogeneity for only two studies included; the conclusion requires further confirmation.

We also analysed CD45RO^+^ T cells, a type of memory T lymphocyte. These T cells are generated upon antigen recognition by the immune system and can survive for months to years thereafter. They are critical for maintaining host defence. CD45RO^+^ T cells are reportedly associated with improved outcomes of several tumours [30–32]. The two included studies that analysed CD45RO^+^ T cells reported a consistently strong relationship between a high density of CD45RO^+^ T cells and OS. The overall HR in this meta-analysis supported this finding, which is also in accordance with the function of memory T cells. Further cohort studies of the impact of CD45RO^+^ T cells on the survival outcomes of EC patients are needed.

The role of regulatory T cells (Tregs) in EC is unclear. This regulatory subset of lymphocytes suppresses the proliferation of effector T cells and cytokine secretion and enables the escape of malignant cells from immunological surveillance. FoxP3 is a marker of Tregs, and a large number of FoxP3^+^ Tregs are related to a poor prognosis of multiple types of tumours [33–35]. In the present meta-analysis, we investigated the relationship of FoxP3^+^ T cells with OS and RFS, but there were no significant conclusions in the pooled HRs. Actually, the three included studies also failed to find a significant relationship, and in one study [26], over half of the EC patients showed no FoxP3^+^ T cells in tumour tissue, suggesting that FoxP3^+^ T cells are not active in the EC tumour microenvironment.

TAMs are an important component of the innate immune system. Immature monocytes are released from the bone marrow and recruited by chemokines to the tumour microenvironment. In there, under the influence of environmental stimuli, TAMs differentiate into M1 (classically activated) or M2 (alternatively activated) macrophages. M1 macrophages kill pathogens and malignant cells, whereas M2 macrophages promote tumour angiogenesis and growth. The predictive role of TAMs in breast, lung, prostate, liver, and gastric cancer is controversial [36–40]. TAMs have been reported to be related to both improved and worsened prognoses, possibly due to the different functions of M1 and M2 macrophages and/or the localisation of TAMs. We found no significant correlation between TAMs and OS. In Ohno et al.'s study [17], only HR-c and HR-e were significantly associated with RFS, whereas HR-f had a trend towards a significant association with RFS. Because there was significant heterogeneity among these studies, subgroup analyses were carried out. Interestingly, only CD163^+^ TAMs (M2) were significantly associated with RFS. This is in accordance with the role of M2 macrophages in promoting EC development. CD68^+^ TAMs were not associated with the outcomes of EC. Because both M1 and M2 macrophages are CD68^+^ TAMs, and each type has an opposite effect on tumours, defining the effects of each of the two kinds of macrophages on RFS is challenging. In addition, the results must be interpreted with caution because only six studies were evaluated. Further clinical research, in which M1 and M2 macrophages are analysed separately, should be conducted. Several studies have focused on CD57^+^ NK cells [18, 23] and DCs [41, 42]. However, these studies used different outcome indices, which hampered the meta-analysis. More research on these immune cells is thus needed.

This study had several strengths. First, we systematically summarised the results of studies on the associations between immune cells and EC outcomes. Second, we used multivariate analysis data, which are more powerful than univariate analysis data. However, this study also had limitations. First, the data of some studies were incomplete, which may affect the results. Second, the small number of included studies reduced the statistical power. Third, we analysed only four types of immune cells because we were unable to find studies of the associations of other immune cells with the prognosis of EC.

## 5. Conclusions

In conclusion, our meta-analysis provides evidence that high CD8^+^ T cell and high CD45RO^+^ T cell densities in tumour were strongly associated with favorable outcomes in EC patients. Additionally, a high TAM density was associated with a worse outcome in EC patients. Because of the small number of included studies, a further study is needed to confirm our conclusions.

## Figures and Tables

**Table 1:** Characteristics of eligible studies.

Author (year)	Ethnicity	*N*	Cell type	Age (year)	Follow-up	Tumour type, FIGO stage	Threshold for positive score	Localisation	Score
Salvesen (1999) [7]	European	60	CD68^+^ TAM	≤66 : 29, >66 : 31	Median, 11 y (8-15)	EC, I-IV	Median, 33 cell/high-power field (HPF)	Hotspot	7
Ohno (2004) [17]	Asian	70	CD68^+^ TAM	Mean, 57.3 (26-78)	Median, 3.28 y (0.15-8.5)	EC, I-IV	Median, nest TAM: 10.7; hotspot TAM: 12.1; stroma TAM, 19.1; margin TAM, 19.3	Hotspot, nest, margin, stroma	7
Giatromanolaki (2008) [26]	European	79	FoxP3^+^ T cell	—	Median, 69 m (6-182)	EEC, I	3–8 cells/×100 optical field	Stroma	6
Soeda (2008) [8]	Asian	76	CD68^+^ TAM	Mean, 55.9 (27-80)	Mean, 82 m (13-130)	EC, I-III	≥20	Margin	5
Ino (2008) [18]	Asian	65	CD8^+^ T cell	Mean, 57.7	Median, 72 m (5-148)	EEC, I-IV	Intraepithelial: CD8^+^ T cell ≥ 25; stromal: CD8^+^T cell ≥ 40	(a) Intraepithelial (b) Stromal or margin	5
de Jong (2009 [25], 2012 [24]), Versluis (2015) [28], Versluis (2017) [27]^∗^	European	368	CD8^+^ T cell CD45RO^+^ T cell	Median, 65 (32-89)	Median 6.2 y (2.4-10.5)	EC, I-IV	CD8^+^ T cell > 4 cells/0.283 mm^2^, CD45RO^+^ T cell: absent	Intratumour	7
Jiang (2012, 2013) [4, 5]^∗^	Asian	186	CD68^+^ TAM	Mean, 58.3 (21-84)	Mean, 76.3 m (5-151)	EEC, I-IV	Mean	2012: margin; 2013: the entire tumour region	7
Kubler (2014) [6]	European	163	CD163^+^ TAM FoxP3^+^ T cell	Median, 68 ± 10.37 (41-95)	Median, 7.92 y (6.92-8.92)	EC, I-IV	Median	Tumour nest	5
Ohnishi (2016) [23]	Asian	79	CD8 T cell	Mean, 59 (30-74)	—	EC, I-IV	≥120 cells/0.028 mm^2^/HPF	Tumour nest	6
Workel (2016) [22]	European	305	CD8 T cell	≤60 : 118, >60 : 187	—	EC, I-IV	>4 cells/0.283 mm^2^	Tumour nest	6
Rashid (2017) [21]	AA; EA	110	FoxP3^+^ T cell CD45RO^+^ T cell CD8^+^ T cell	Median, AA : 61.46, EA : 63.55	Mean, AA : 44.74 m, EA : 59.02 m	—	FoxP3^+^ T cell &CD8^+^ T cell > 2.73; CD45RO^+^ T cell > 22.2	Tumour stroma	6
Yamashita (2018) [19]	Asian	149	CD8 T cell	<60 : 76, ≥60 : 73	Median, 38 m (1-134)	EC, I-IV	>30% staining	The entire tumour region	6
Kim (2018) [20]	Asian	183	CD8 T cell	Mean, 53.0 ± 10.4	Median, 30.3	EC, I-IV	>21 cells/0.237 mm^2^/HPF	Center of tumour	7

TAM = tumour-associated macrophage; EC = endometrial carcinoma; EEC = endometrial endometrioid carcinoma; y = year; m = month; score = NOS score; AA = African America; EA = European American. ^∗^Data from studies of the same populations were only extracted once.

**Table 2:** Meta-analysis of immune cells and prognostic indices in EC patients.

Group	Study number	Number of case	Pooled result, HR (95% CI)	PZ value	PH value	*I* ^2^
CD8-OS	5	740	0.201 (0.076-0.533)	**0.001**	0.104	45.2%
CD8-PFS-a/b	3	397	0.308 (0.139-0.683)/0.306 (0.137-0.687)	**0.004**	0.555/0.554	0.0%
CD8-DSS	2	673	0.366 (0.222-0.604)	**0.000**	0.353	0.0%
CD8-DFS	2	673	0.466 (0.329-0.660)	**0.000**	0.396	0.0%
FoxP3^+^ T cell-OS	3	352	1.264 (0.419-3.812)	0.678	0.212	35.6%
FoxP3^+^ T cell-RFS	2	273	1.000 (0.995-1.005)	0.999	0.655	0.0%
CD45RO^+^ T cell-OS	2	478	0.415 (0.241-0.714)	**0.001**	0.638	0.0%
TAM-OS	3	425	1.985 (0.771-5.113)	0.156	0.628	0.0%
TAM-RFS-c/d	4	479	3.423 (1.289-9.094)/1.770 (0.458-6.843)	**0.014**/0.408	0.364/0.068	5.9%/57.8%
TAM-RFS-e/f	4	479	2.369 (1.003-5.598)/2.258 (0.966-5.282)	**0.049**/0.06	0.403/0.373	0.0%/3.9%

PZ: *p* value of the *Z*-test; PH: *p* value of the heterogeneity test.

**Table 3:** Subgroup analysis of immune cells and prognostic indices in EC patients.

Group	Subgroup	Study number	Number of case	Pooled result, HR (95% CI)	PZ value	PH value	*I* ^2^
CD8-OS	Intraepithelial	3	630	0.320 (0.120-0.849)	**0.002**	0.265	24.8%
	Stromal	1^∗^	110	0.064 (0.016-0.253)	**0.001**	0.915	0.0%
	Whole^#^	1	149	0.850 (0.070-10.560)	0.899	—	—

CD8-PFS	Intraepithelial	2	248	0.262 (0.111-0.621)	**0.002**	0.612	0.0%
	Stromal	1	65	0.460 (0.040-5.220)	0.532	—	—
	Whole^#^	1	149	0.790 (0.100-6.440)	0.824	—	—

FoxP3^+^ T cell-OS	Intraepithelial	1	163	1.000 (1.000-1.000)	0.678	—	—
	Stromal	2	189	1.264 (0.419-3.812)	0.678	0.212	35.6%

FoxP3^+^ T cell-RFS	Intraepithelial	1	163	1.000 (1000-1.010)	1.000	—	—
	Stromal	1^∗^	110	1.103 (0.516-2.360)	0.801	0.377	0.0%

CD45RO^+^ T cell-OS	Intraepithelial	1	368	0.490 (0.260-0.950)	**0.031**	—	—
	Stromal	1∗	110	0.280 (0.103-0.757)	**0.012**	0.833	0.0%

TAM-OS	CD68^+^ TAM	2	262	3.219 (0.740-13.998)	0.119	0.637	0.0%
	CD163^+^ TAM	1	163	1.400 (0.410-4.860)	0.586	—	—
	FIGO stage I-III	1	76	1.810 (0.109-29.996)	0.679	—	—
	FIGO stage I-IV	2	349	2.008 (0.735-5.487)	0.174	0.336	0.0%

TAM-RFS-c/d	Intraepithelial	3	293	3.582 (0.889-14.264)/1.397 (0.260-7.508)	0.070/0.696	0.210/0.052	36%/66.2%
	Stromal	1	186	4.200 (1.900–99.800)	0.156	—	—
	CD68^+^ TAM	3	316	2.627 (0.858-8.049)/1.104 (0.293-4.156)	0.091/0.883	0.343/0.159	6.5%/45.7%
	CD163^+^ TAM	1	163	8.310 (1.030-67.300)	**0.035**	—	—

TAM-RFS-e/f	Intraepithelial	2	223	2.836 (0.431-18.676)	0.278	0.127	57.0%
	Stromal	2	256	2.277 (0.665-7.792)/2.056 (0.651-6.494)	0.179/0.219	0.439/0.385	0.0%
	CD68^+^ TAM	3	316	1.766 (0.679-4.590)/1.689 (0.676-4.222)	0.243/0.262	0.603/0.588	0.0%
	CD163^+^ TAM	1	163	8.310 (1.030-67.300)	**0.035**	—	—

PZ: *p* value of the *Z*-test; PH: *p* value of the heterogeneity test. ^∗^One study included two groups. ^#^Study calculated immune cells in the whole tumour tissue.

## Data Availability

The data used to support the findings of this study are available from the corresponding author upon request.

## Supplementary Materials

Supplementary Materials PRISMA 2009 Checklist Table S1: HRs and 95% CIs extracted or calculated from each included study. Figure S1: forest plots of subgroup analysis between CD8^+^ T cell and survival prognosis of EC patients. A study by Ino et al. reported two Kaplan–Meier curves according to the immune cell localisation in tumour tissue; we calculated the HR and 95% CI of that study separately. Figure S2: forest plots of subgroup analysis between FoxP3^+^ T cell and survival prognosis of 307 EC patients. Figure S3: forest plots of subgroup analysis between CD45RO^+^ T cell and survival prognosis of EC patients. Figure S4: (A) forest plots of subgroup analysis according to the location of immune cells in tumour tissue between TAM and survival prognosis of EC patients. (B) Forest plots of subgroup analysis according to immune cell markers between TAM and survival prognosis of EC patients. A study by “Satoshi (2004)” reported four Kaplan–Meier curves according to the immune cell localisation in tumour tissue; we calculated the HR and 95% CI of that study separately. Figure S5: sensitive analysis was conducted to assess the effect of a specific study on a pooled HR for CD8^+^ T cell by omitting one study at a time. (A) CD8^+^ T cell with OS; (B) CD8^+^ T cell with PFS; (C) CD8^+^ T cell with DFS; (D) CD8^+^ T cell with DSS. Figure S6: sensitive analysis was conducted to assess the effect of a specific study on a pooled HR for CD45RO^+^ T cell, FOXP3^+^ T cell, and TAM by omitting one study at a time. (A) CD45RO^+^ T cell with OS; (B) FOXP3^+^ T cell with OS; (C) FOXP3^+^ T cell with RFS; (D) TAM with OS; (E) TAM with PFS.
