# Supplementary material for: Tissue Infiltrating Immune Cells as Prognostic Biomarkers in Endometrial Cancer: A Meta-Analysis
Source: Dis Markers. 2020 Jan 28;2020:1805764. doi: 10.1155/2020/1805764 (PMC7008300; doi:10.1155/2020/1805764)
Supplement: Supplementary Materials — PRISMA 2009 Checklist Table S1: HRs and 95% CIs extracted or calculated from each included study. Figure S1: forest plots of subgroup analysis between CD8+ T cell and survival prognosis of EC patients. A study by Ino et al. reported two Kaplan–Meier curves according to the immune cell localisation in tumour tissue; we calculated the HR and 95% CI of that study separately. Figure S2: forest plots of subgroup analysis between FoxP3+ T cell and survival prognosis of 307 EC patients. Figure S3: forest plots of subgroup analysis between CD45RO+ T cell and survival prognosis of EC patients. Figure S4: (A) forest plots of subgroup analysis according to the location of immune cells in tumour tissue between TAM and survival prognosis of EC patients. (B) Forest plots of subgroup analysis according to immune cell markers between TAM and survival prognosis of EC patients. A study by “Satoshi (2004)” reported four Kaplan–Meier curves according to the immune cell localisation in tumour tissue; we calculated the HR and 95% CI of that study separately. Figure S5: sensitive analysis was conducted to assess the effect of a specific study on a pooled HR for CD8+ T cell by omitting one study at a time. (A) CD8+ T cell with OS; (B) CD8+ T cell with PFS; (C) CD8+ T cell with DFS; (D) CD8+ T cell with DSS. Figure S6: sensitive analysis was conducted to assess the effect of a specific study on a pooled HR for CD45RO+ T cell, FOXP3+ T cell, and TAM by omitting one study at a time. (A) CD45RO+ T cell with OS; (B) FOXP3+ T cell with OS; (C) FOXP3+ T cell with RFS; (D) TAM with OS; (E) TAM with PFS. [file 1805764.f1.docx]

**Supplementary Material**


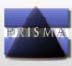
**PRISMA 2009 Checklist**

| **Section/topic** | | **#** | | **Checklist item** | | **Reported on page #** |
| --- | --- | --- | --- | --- | --- | --- |
| **TITLE** | | | | | |  |
| Title | | 1 | | The report is identified as a meta-analysis. | | 1 |
| **ABSTRACT** | | | | | |  |
| Structured summary | | 2 | | The structured abstract including background; methods, results. conclusion. | | 1 |
| **INTRODUCTION** | | | | | |  |
| Rationale | | 3 | | Described in introduction. | | 1, 2 |
| Objectives | | 4 | | Stated in introduction. | | 2 |
| **METHODS** | | | | | |  |
| Protocol and registration | | 5 | | The protocol is described in the Materials and Methods. Registration does not apply. | | 2 |
| Eligibility criteria | | 6 | | Described in Search Strategy and Inclusion Criteria. | | 2 |
| Information sources | | 7 | | Described in Search Strategy and Inclusion Criteria. | | 2 |
| Search | | 8 | | Described in Search Strategy and Inclusion Criteria. | | 2 |
| Study selection | | 9 | | Described in Search Strategy and Inclusion Criteria. | | 2 |
| Data collection process | | 10 | | Described in Data Extraction and Quality Assessment. | | 2 |
| Data items | | 11 | | Described in Data Extraction and Quality Assessment. | | 2 |
| Risk of bias in individual studies | | 12 | | Described in Statistical Analysis. | | 2 |
| Summary measures | | 13 | | Described in Statistical Analysis. | | 2 |
| Synthesis of results | | 14 | | Described in Statistical Analysis. | | 2 |
| **Section/topic** | **#** | | **Checklist item** | | **Reported on page #** | |
| Risk of bias across studies | 15 | | Described in Statistical Analysis. | | 2 | |
| Additional analyses | 16 | | Described in Statistical Analysis. | | 2 | |
| **RESULTS** | | | | |  | |
| Study selection | 17 | | Described in literature Search and Characteristics of Eligible Studies, and flow diagram was shown in Figure 1. | | 2,4 | |
| Study characteristics | 18 | | Described in literature Search and Characteristics of Eligible Studies, and characteristics were shown in Table 1 and Table S1. | | 2-4 | |
| Risk of bias within studies | 19 | | Described in literature Search and Characteristics of Eligible Studies, and Table 1 and Table S1. | | 2-4 | |
| Results of individual studies | 20 | | Simple summary data for each intervention group and effect estimates and confidence intervals were shown in Figure 2-4. | | 5,7,8 | |
| Synthesis of results | 21 | | Results of each meta-analysis were shown in Table 2. | | 4 | |
| Risk of bias across studies | 22 | | Risk of bias across studies was shown in Publication Bias Analysis and Sensitivity Analysis. | | 6 | |
| Additional analysis | 23 | | sensitivity or subgroup analyses were shown in Publication Bias Analysis and Sensitivity Analysis and Table 3. | | 6 | |
| **DISCUSSION** | | | | |  | |
| Summary of evidence | 24 | | Summary was shown in Discussion. | | 6 | |
| Limitations | 25 | | Discussed in Discussion. | | 6-9 | |
| Conclusions | 26 | | Discussed in Conclusions. | | 9 | |
| **FUNDING** | | | | |  | |
| Funding | 27 | | Described in Funding Statement. | | 9 | |

*From:*  Moher D, Liberati A, Tetzlaff J, Altman DG, The PRISMA Group (2009). Preferred Reporting Items for Systematic Reviews and Meta-Analyses: The PRISMA Statement. PLoS Med 6(7): e1000097. doi:10.1371/journal.pmed1000097

For more information, visit: **www.prisma-statement.org**.

**Table S1.** HRs and 95%CIs extracted or calculated from each included study.

| **Study** | **Outcome** | **Location** | **HR(95%CI)** | **Study** | **Outcome** | **Location** | **HR(95%CI)** |
| --- | --- | --- | --- | --- | --- | --- | --- |
| **CD8^+^T cell(reference=low CD8 density)** | |  |  | **FoxP3^+^ T cell (reference=low FoxP3+ T cell density)** | | |  |
| R.A. (2009) | OS | intraepithelial | 0.48 (0.260–0.890) | Alexandra(2008) | OS | stromal | 4.70 (0.79-27.86)* |
| Koji (2016) | OS | intraepithelial | 0.21 (0.010-4.880)* | Kirsten(2014) | OS | intraepithelial | 1.00 (1.00–1.00) |
| Tariq(2017)-group-AA^#^ | OS | stromal | 0.06 (0.010-0.340)* | Tariq(2017)-group-AA | OS | stromal | 0.58 (0.11-3.58)* |
| Tariq(2017)-group-EA | OS | stromal | 0.07 (0.010-0.850)* | Tariq(2017)-group-EA | OS | stromal | 0.93(0.29-3.05)* |
| Hitomi(2018) | OS | whole | 0.85 (0.070-10.560)* |  |  |  |  |
| Jisup (2018) | OS | intraepithelial | 0.095 (0.014–0.650) | Kirsten(2014) | RFS | intraepithelial | 1.00 (1.00–1.01) |
|  |  |  |  | Tariq(2017)-group-AA | RFS | stromal | 0.82 (0.30-2.24)* |
| Kazuhiko(2008) | PFS | intraepithelial/ | 0.440 (0.050-3.920)*^a^/ | Tariq(2017)-group-EA | RFS | stromal | 1.64 (0.51-5.22)* |
|  | PFS | stromal | 0.460 (0.040-5.220)*^b^ | **TAM (reference=low TAM density)** | |  |  |
| Hitomi(2018) | PFS | whole | 0.790 (0.10-6.440)* | Shu(2008) | OS | stromal | 1.81 (0.11-30.21)* |
| Jisup(2018) | PFS | intraepithelial | 0.238 (0.093–0.608) | X.F.J.(2013) | OS | whole | 4.0 (3.1–97.7) |
|  |  |  |  | Kirsten(2014) | OS | intraepithelial | 1.4(0.41-4.86) |
| R.A. (2012) | DSS | intraepithelial | 0.320 (0.180-0.570) | Helga(1999) | RFS | intraepithelial | 1.2 (0.27–5.60) |
| Hagma(2016） | DSS | intraepithelial | 0.557 (0.201-1.542) | SATOSHI(2004) | RFS | intraepithelial | 8.33 (0.77-90.66)*^c^/ 0.35 (0.07-1.78)*^d^ |
| R.A.(2012) | DFS | intraepithelial | 0.420 (0.270-0.630) |  |  | stromal | 1.55 (0.32-7.39)*^e^/1.43 (0.35-5.90)*^f^ |
| Hagma(2016) | DFS | intraepithelial | 0.579 (0.315-1.063) | X.F.J. (2012) | RFS | stromal | 4.2 ( 1.9–99.8) |
| **CD45RO^+^ T cell (reference=low CD45RO+ T cell density)** | | |  | Kirsten(2014) | RFS | intraepithelial | 8.31(1.03-67.30) |
| R.A.(2009) | OS | intraepithelial | 0.49 (0.26-0.95)* |  |  |  |  |
| Tariq(2017)-group-AA | OS | stromal | 0.25 (0.06-1.07)* |  |  |  |  |
| Tariq(2017)-group-EA | OS | stromal | 0.31 (0.08-1.26)* |  |  |  |  |

Note: *, HR and 95% CIs were calculated by Engauge Digitizer software from Kaplan–Meier survival curve provided by each study. #, Study "Tariq (2017)" reported two group according to different ethnicity, we calculated the HR(95%CI) from the two groups, respectively. AA, African America; EA, European American. a/b, study “Kazuhiko (2008)” reported two Kaplan–Meier curves according to the immune cell localisation in tumour tissue, we calculated the HR and 95%CI of that study separately. c-f, Study "SATOSHI (2004)" reported four Kaplan–Meier curves according to the immune cell localisation in tumour tissue, we calculated the HR and 95%CI of that study separately.

## Supplementary Figures

**Figure S1** Forest plots of subgroup analysis between CD8^+^ T cell and survival prognosis of EC patients. Study “Kazuhiko (2008)” reported two Kaplan–Meier curves according to the immune cell localisation in tumour tissue, we calculated the HR and 95%CI of that study separately.

**Figure S2** Forest plots of subgroup analysis between FoxP3^+^ T cell and survival prognosis of EC patients.

**Figure S3** Forest plots of subgroup analysis between CD45RO^+^ T cell and survival prognosis of EC patients.

**Figure S4** **A**. Forest plots of subgroup analysis according to location of immune cells in tumor tissue between TAM and survival prognosis of EC patients. **B**. Forest plots of subgroup analysis according to immune cell markers between TAM and survival prognosis of EC patients. Study "**SATOSHI (2004)**" reported four Kaplan–Meier curves according to the immune cell localisation in tumour tissue, we calculated the HR and 95%CI of that study separately.

A

B

C

D

**Figure S5.** Sensitive analysis was conducted to assess the effect of a specific study on pooled HR for CD8^+^ T cell by omitting one study at a time. (A) CD8^+^ T cell with OS (B) CD8^+^ T cell with PFS (C) CD8^+^ T cell with DFS (D) CD8^+^ T cell with DSS.

A

C

B

D

E

**Figure S6.** Sensitive analysis was conducted to assess the effect of a specific study on pooled HR for CD45RO^+^ T cell, FOXP3^+^ T cell and TAM by omitting one study at a time. (A) CD45RO^+^ T cell with OS (B) FOXP3^+^ T cell with OS (C) FOXP3^+^ T cell with RFS (D) TAM with OS (E) TAM with PFS.
